# Fibrinogen Replacement Therapy for Traumatic Coagulopathy: Does the Fibrinogen Source Matter?

**DOI:** 10.3390/ijms22042185

**Published:** 2021-02-22

**Authors:** Gael B. Morrow, Molly S. A. Carlier, Sruti Dasgupta, Fiona B. Craigen, Nicola J. Mutch, Nicola Curry

**Affiliations:** 1 Radcliffe Department of Medicine, University of Oxford, Oxford OX3 9DU, UK; gael.morrow@ndcls.ox.ac.uk; 2 Aberdeen Cardiovascular & Diabetes Centre, School of Medicine, Medical Sciences and Nutrition, Institute of Medical Sciences, University of Aberdeen, Aberdeen AB25 2ZD, UK; molly.carlier@abdn.ac.uk (M.S.A.C.); sruti.dasgupta@abdn.ac.uk (S.D.); fiona.craigen@abdn.ac.uk (F.B.C.); n.j.mutch@abdn.ac.uk (N.J.M.); 3 Oxford Haemophilia & Thrombosis Centre, NIHR Oxford Biomedical Research Centre, Oxford University Hospitals NHS Foundation Trust, Oxford OX3 7LE, UK

**Keywords:** fibrinogen, cryoprecipitate, trauma coagulopathy, α_2_-antiplasmin, factor XIII

## Abstract

Fibrinogen is the first coagulation protein to reach critically low levels during traumatic haemorrhage. There have been no differential effects on clinical outcomes between the two main sources of fibrinogen replacement: cryoprecipitate and fibrinogen concentrate (Fg-C). However, the constituents of these sources are very different. The aim of this study was to determine whether these give rise to any differences in clot stability that may occur during trauma haemorrhage. Fibrinogen deficient plasma (FDP) was spiked with fibrinogen from cryoprecipitate or Fg-C. A panel of coagulation factors, rotational thromboelastography (ROTEM), thrombin generation (TG), clot lysis and confocal microscopy were performed to measure clot strength and stability. Increasing concentrations of fibrinogen from Fg-C or cryoprecipitate added to FDP strongly correlated with Clauss fibrinogen, demonstrating good recovery of fibrinogen (r^2^ = 0.99). A marked increase in Factor VIII, XIII and α_2_-antiplasmin was observed in cryoprecipitate (*p* < 0.05). Increasing concentrations of fibrinogen from both sources were strongly correlated with ROTEM parameters (r^2^ = 0.78–0.98). Cryoprecipitate therapy improved TG potential, increased fibrinolytic resistance and formed more homogeneous fibrin clots, compared to Fg-C. In summary, our data indicate that cryoprecipitate may be a superior source of fibrinogen to successfully control bleeding in trauma coagulopathy. However, these different products require evaluation in a clinical setting.

## 1. Introduction

Trauma is the leading cause of preventable deaths worldwide [1], and 40% of deaths due to injury are a result of uncontrolled bleeding or its consequences [2]. Death from haemorrhage is frequently early, with around 60% of deaths occurring within the first 3 h of injury [3]. Management and transfusion requirements have changed dramatically over the past two decades and have primarily been driven by an increased understanding of the pathology of trauma-induced coagulopathy (TIC).

TIC is a multi-phenotypic disease state that comprises disorders of coagulation and inflammation, and it describes the overall failure of the coagulation system to maintain haemostasis after major injury. It is characterised by impaired clot formation and breakdown, alongside overall vascular homeostasis. TIC is associated with significantly poorer outcomes, including increased need for major haemorrhage therapy and early transfusion requirements, development of organ failure and 3–4-fold increased risk of death [4,5,6,7].

Fibrinogen, the key pro-coagulant factor required for stable clot formation, is the first coagulation protein to reach critically low levels during traumatic haemorrhage [8,9,10]. Fibrinogen is cleaved by thrombin to insoluble fibrin, which forms a haemostatic plug at sites of bleeding. Fibrinogen can also activate the integrin α_IIb_β_3_ on the platelet surface, resulting in degranulation and further amplification of primary haemostasis [11,12,13]. Fibrin is a viscoelastic polymer, and its properties are crucial in determining the physical and mechanical characteristics of the clot [14]. Fibrin cross-linking by the transglutaminase enzyme, activated factor XIII (FXIIIa), occurs between neighbouring fibrin molecules to enhance clot stability against mechanical stress [15,16]. Alpha 2-antiplasmin (α_2_AP) is also cross-linked to fibrin via FXIIIa to stabilise the clot against premature degradation by plasmin [17,18].

Therefore, there is rationale that early fibrinogen replacement may be an effective therapy for major trauma haemorrhage [19,20,21]. There are two main sources of fibrinogen replacement: cryoprecipitate and fibrinogen concentrate (Fg-C). Cryoprecipitate is a pooled blood component derived from whole blood donation and has a variable but high fibrinogen concentration (8–16 g/L) [22]. Additionally, cryoprecipitate is rich in a number of other coagulation factors that are not present in Fg-C [23]. These include pro-coagulant factors, in particular factor VIII (FVIII), that will support thrombin generation and anti-fibrinolytic factors, such as α_2_AP and FXIII. Fg-C has a standard dose of 20 g/L and currently is not licensed in the UK for acquired bleeding. However, Fg-C has been used for many years to prevent bleeding in inherited dysfibrinogenaemia and hypofibrinogenaemia and has a favourable safety profile [24].

The aim of this study is to determine differences in clot strength and stability between cryoprecipitate and Fg-C preparations using a range of laboratory tests, including fibrinogen recovery, thrombin generation (TG), rotational thromboelastography (ROTEM) and measures of fibrinolysis. Studies comparing clinical outcomes have found no differential effects between Fg-C and cryoprecipitate; however, the constituents of these products are very different and may alter clot stability during trauma haemorrhage.

## 2. Results

Preliminary experiments determined the concentration of coagulation factors in pooled normal plasma (PNP), fibrinogen deficient plasma (FDP), cryoprecipitate and Fg-C (Table 1). The following coagulation factors were measured: factors II, V, VII, VIII, IX, X, XI, XII, XIII, von Willebrand factor (vWF) and α_2_AP. As expected, fibrinogen was not detected in FDP, and FV, FVIII and FXIII levels were also lower than PNP due to the method of manufacture (*p* < 0.01; Table 1). Cryoprecipitate is prepared by controlled thawing of plasma to precipitate high molecular weight proteins; therefore, it was expected that fibrinogen, FVIII and vWF levels would be significantly higher than PNP (*p* < 0.05; Table 1). α_2_AP levels were 98-fold higher in cryoprecipitate than in Fg-C (*p* < 0.001). The only coagulation factors detected in Fg-C were fibrinogen, vWF and FVIII (Table 1).

**Figure 1:**
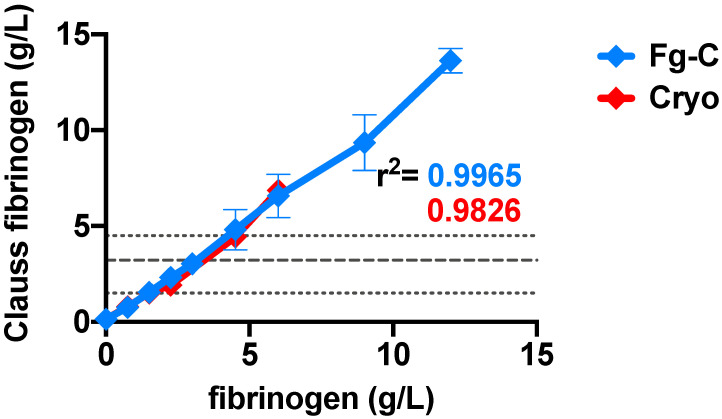
Fibrinogen dose response in fibrinogen concentrate and cryoprecipitate strongly correlates with Clauss fibrinogen.

**Figure 2:**
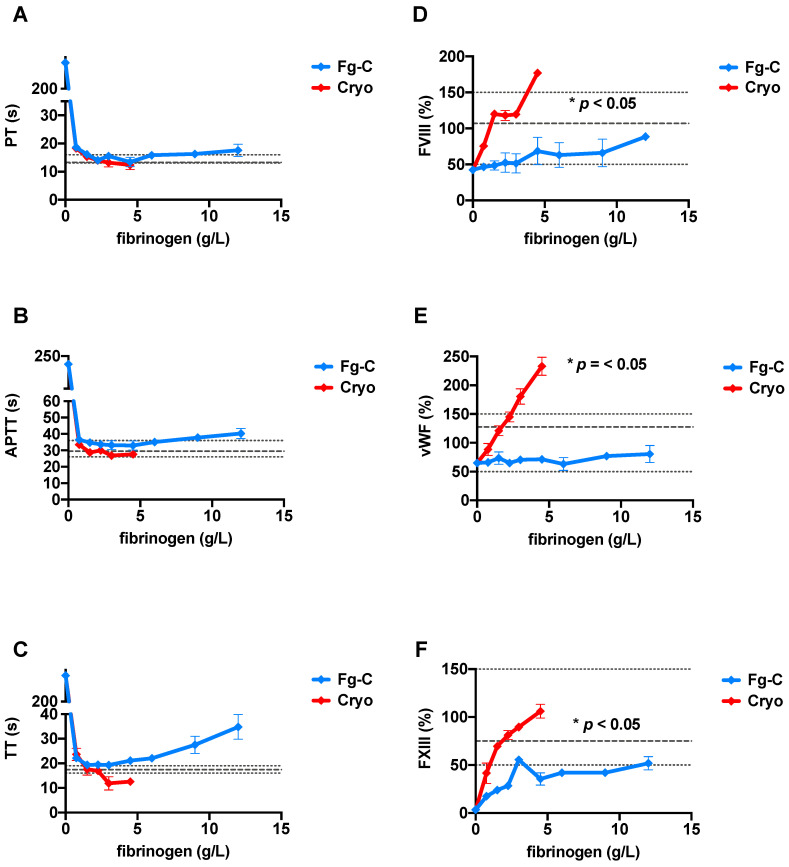
Clotting time tests and coagulation factors VIII, XIII and von Willebrand factor are more sensitive to increasing concentrations of cryoprecipitate than fibrinogen concentrate. Increasing concentrations of fibrinogen from either fibrinogen concentrate (Fg-C—blue) or cryoprecipitate (cryo—red) were added to fibrinogen deficient plasma (FDP) and (**A**) prothrombin time (PT), (**B**) activated partial thromboplastin time (APTT) and (**C**) thrombin time (TT) measured using a Sysmex CS-5100 haematology analyser. *n* = 2. Increasing concentrations of fibrinogen from either fibrinogen concentrate (Fg-C—blue) or cryoprecipitate (cryo—red) were added to fibrinogen deficient plasma (FDP), and (**D**) FVIII, (**E**) von Willebrand factor (vWF) and (**F**) FXIII were measured using a Sysmex CS-5100 haematology analyser. Normal ranges (short dash) and PNP (long dash) are shown by grey dotted lines. Statistical significance is denoted on each relevant graph. Data are represented as mean ± SD. * *p* < 0.05. *n* = 2, assays performed in duplicate.

Increasing concentrations of exogenous fibrinogen from Fg-C or cryoprecipitate added to FDP strongly correlated with the Clauss fibrinogen level, demonstrating good recovery of fibrinogen in both sources (r^2^ = 0.99 and 0.98 for Fg-C and cryoprecipitate, respectively *p* < 0.0001; Figure 1). Standard clotting tests, prothrombin time (PT), activated partial thromboplastin time (APTT) and thrombin time (TT) shortened from ≥240 s (with no fibrinogen present) to within the normal range as fibrinogen concentration increased from both Fg-C and cryoprecipitate sources (Figure 2A–C). The fibrinogen concentration required to bring the PT, APTT and TT into the normal range was consistently lower for cryoprecipitate than Fg-C; 1.5 vs. 2.25 g/L, 0.75 vs. 1.5 g/L and 1.5 vs. 3 g/L, respectively (Figure 2A–C). At higher concentrations of Fg-C (≥4.5 g/L), there was a progressive prolongation of all standard clotting times (Figure 2A–C). Most strikingly, TT did not shorten to within the normal range with Fg-C (Figure 2C). Both PT and APTT showed similar, albeit less dramatic, lengthening of clotting times with Fg-C ≥ 4.5 g/L (Figure 2C).

Increasing concentrations of fibrinogen from either fibrinogen concentrate (Fg-C- blue) or cryoprecipitate (cryo- red) were added to fibrinogen deficient plasma (FDP) (x-axis), and Clauss fibrinogen (y-axis) was measured using a Sysmex CS-5100 haematology analyser. There was a strong correlation between both Fg-C and cryoprecipitate and Clauss fibrinogen; r^2^ = 0.99 and 0.98, respectively. *p* < 0.0001. Data are represented as mean ± SD. Normal ranges (short dash) and PNP (long dash) are shown by grey dotted lines. *n* = 2, assays performed in duplicate.

To determine whether any of the coagulation factors present in cryoprecipitate may influence the standard clotting times, a panel of coagulation factor assays was performed (Figure 2). PT-based factors (extrinsic- FII, FV, VII and X) and APTT-based factors (intrinsic- IX and XI) were not significantly different across varying fibrinogen concentrations from both Fg-C and cryoprecipitate sources (data not shown). However, a marked increase in FVIII, FXIII and vWF was observed at higher concentrations of cryoprecipitate when compared to Fg-C (Figure 2). This increase was statistically significant (*p* < 0.05).

**Figure 3:**
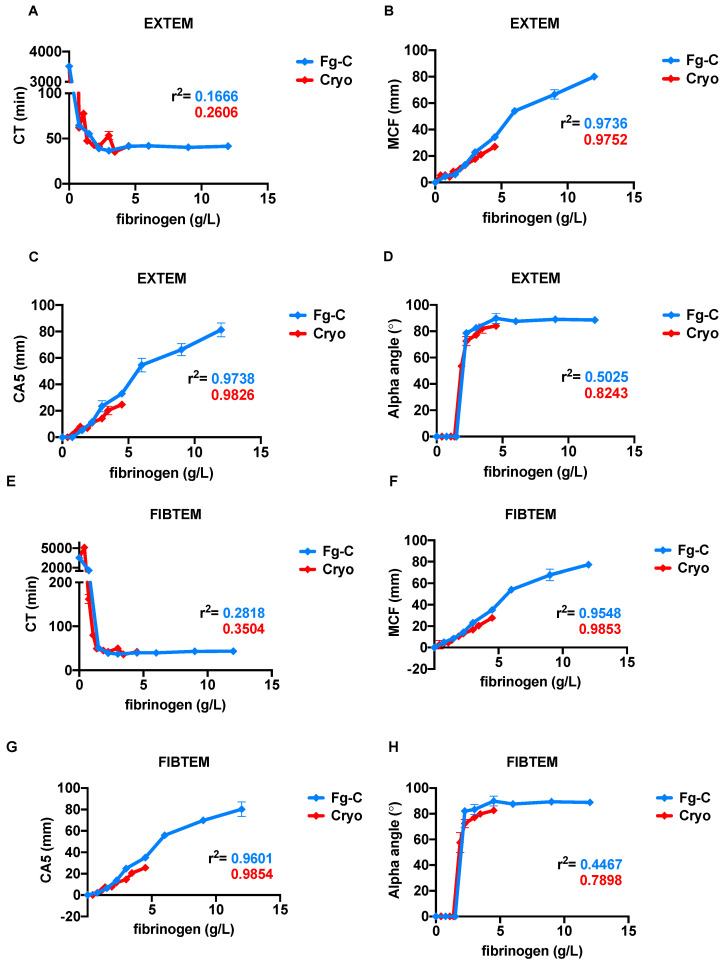
Increasing fibrinogen concentration in cryoprecipitate and fibrinogen concentrate strongly correlates with rotational thromboelastography (ROTEM) parameters. Increasing concentrations of fibrinogen from either fibrinogen concentrate (Fg-C—blue) or cryoprecipitate (cryo—red) were added to fibrinogen deficient plasma (FDP) and ROTEM tests performed. (**A**,**E**) Clotting time (CT), (**B**,**F**) maximum clot firmness (MCF), (**C**,**G**) clot amplitude at 5 min (CA5) and (**D**,**H**) alpha angle parameters are shown for EXTEM and FIBTEM tests, respectively. The r^2^ of correlation for each parameter is depicted on the graph. Data are represented as mean ± SD. *n* = 2, assays performed in triplicate.

ROTEM tests were performed to provide further insight into the kinetics of clots formed from Fg-C and cryoprecipitate (Figure 3). As expected, the ROTEM results for EXTEM and FIBTEM tests were strongly correlated with fibrinogen concentration (Figure 3). EXTEM clotting times (CT) rapidly decreased with rising fibrinogen levels from both cryoprecipitate and Fg-C sources and achieved a CT value within the normal range at 0.75 g/L fibrinogen (Figure 3A). The maximum clot firmness (MCF) and clot amplitude at 5 min (CA5) were positively correlated with the fibrinogen concentration for Fg-C (r^2^ = 0.97; *p* < 0.0001) and cryoprecipitate (r^2^ = 0.97, 0.98; *p* < 0.0001) and were within the normal range at 3.3 and 3.9 g/L fibrinogen, respectively (Figure 3B,C). Higher concentrations of cryoprecipitate were required to normalise the alpha angle compared to Fg-C; 2 vs. 4 g/L, respectively (Figure 3D). Similar results were obtained for the parameters in the FIBTEM test (Figure 3E–H). However, there was no statistical difference between either fibrinogen source for any of the EXTEM or FIBTEM parameters (Figure 3).

**Figure 4:**
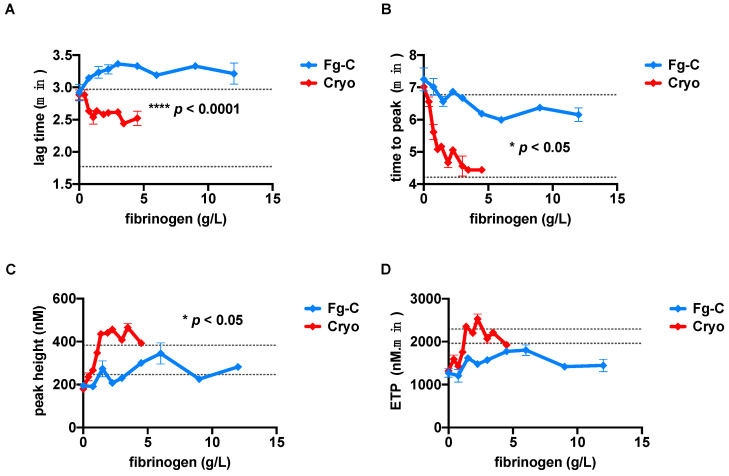
Increasing concentrations of cryoprecipitate, but not fibrinogen concentrate, enhances the thrombin generation potential. Increasing concentrations of fibrinogen from either fibrinogen concentrate (Fg-C—blue) or cryoprecipitate (cryo—red) were added to fibrinogen deficient plasma (FDP) and thrombin generation tests performed. (**A**) Lag time (**B**) time to peak, (**C**) peak height and (**D**) endogenous thrombin potential (ETP) are shown. Levels of statistical significance are depicted on each relevant graph. Normal ranges are shown by grey dotted lines. Data are represented as mean ± SD. * *p* < 0.05, **** *p* < 0.0001. *n* = 2, assays performed in triplicate.

TG experiments were implemented to determine whether there was a difference between cryoprecipitate and Fg-C in their ability to generate thrombin (Figure 4). Increasing concentrations of fibrinogen in cryoprecipitate, but not Fg-C, resulted in shortening of the lag time (*p* < 0.0001; Figure 4A). The time to peak was shortened by both sources with increasing fibrinogen concentration, but cryoprecipitate shortened the time to peak significantly faster than Fg-C, where only a slight improvement was observed (*p* < 0.05; Figure 4B). The peak height and endogenous thrombin potential (ETP) both increased with rising concentrations of fibrinogen from cryoprecipitate (Figure 4C,D). Similar to the lag time, the results for peak height and ETP were not influenced by increasing concentrations of Fg-C (Figure 4C,D). This was expected due to the lack of other coagulation factors in Fg-C, namely FV and FVIII, which fundamentally influence TG. At a fibrinogen concentration of 3 g/L, the lag time, time to peak and peak height, but not ETP, were significantly improved in cryoprecipitate compared to Fg-C (*p* < 0.0001, *p* < 0.05 and *p* < 0.05, respectively; Figure 4A–D).

**Figure 5:**
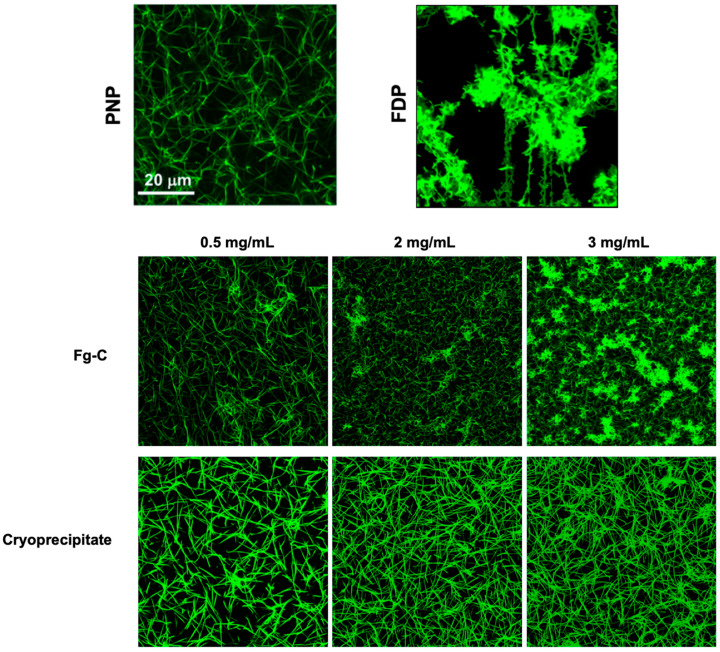
The fibrin network of thrombi formed from cryoprecipitate are more homogeneous than those formed from fibrinogen concentrate. Clots were formed from 30% PNP or FDP and spiked with 0.5, 2 or 3 mg/mL cryoprecipitate or fibrinogen concentrate (Fg-C). Clots were imaged using a ×63 1.4 oil immersion objective and Zeiss 710 laser scanning confocal microscope. Representative image of *n* = 3.

Confocal microscopy imaging of clots formed from FDP spiked with either cryoprecipitate or Fg-C revealed dramatic differences in fibrin clot structure with the different fibrinogen sources (Figure 5). Clots formed with cryoprecipitate demonstrated a fibrin fibre structure similar to that of the PNP control, and increasing the cryoprecipitate concentration produced a denser fibrin network (Figure 5). In contrast, the clots formed with Fg-C showed thinner fibrin fibres and were less homogeneous compared to those formed from cryoprecipitate (Figure 5). Interestingly, at a higher concentration of 2 and 3 mg/mL clusters of fibrin(ogen) were observed within the clot formed from Fg-C, but not cryoprecipitate (Figure 5).

**Figure 6:**
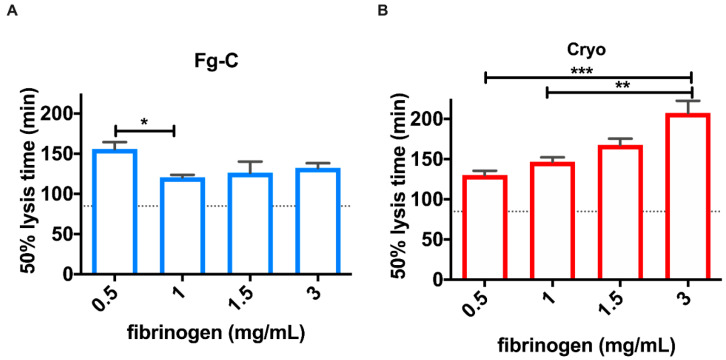
Increasing fibrinogen concentration in cryoprecipitate, but not fibrinogen concentrate, increases clot stability. Clots were formed from 30% FDP and 300 pM tissue plasminogen activator (tPA) spiked with 0.5, 1, 1.5 or 3 mg/mL fibrinogen concentrate (Fg-C; (**A**)) or cryoprecipitate (cryo; (**B**)). Clot formation and lysis were monitored by measuring absorbance at 405 nM every 60 s for 4 h and 50 % lysis times were calculated using the Shiny App software for clot lysis [25]. Dotted lines on the y axis represent the 50% lysis time for control samples (PNP). Data are represented as mean ± SD. * *p* < 0.05, ** *p* < 0.01, *** *p* < 0.001. *n* = 6.

Clots formed from cryoprecipitate showed increased stability against fibrinolytic degradation by exogenous tissue plasminogen activator (tPA) compared to those formed from Fg-C (Figure 6). Increasing fibrinogen concentration with cryoprecipitate prolonged the lysis time (*p* < 0.001), whereas increasing Fg-C did not alter lysis times (Figure 6). However, addition of fibrinogen from either fibrinogen source delayed the lysis time at all concentrations of fibrinogen when compared to the control (PNP; Figure 6). At 1.5 mg/mL, fibrinogen clot lysis times were delayed 1.9- and 1.5-fold, when compared to PNP, for cryoprecipitate and Fg-C, respectively (Figure 6).

## 3. Discussion

Normal haemostasis is critically dependent on fibrinogen as it is critical for stable blood clot formation [26,27]. While fibrinogen levels are the first to be depleted during massive haemorrhage [8,28], there is limited evidence to support a specific effective fibrinogen concentration during active bleeding [29]. Fibrinogen can be replaced using fresh frozen plasma (FFP), cryoprecipitate or Fg-C, all of which have varying concentrations of fibrinogen (2 g/L, 8–16 g/L and 20 g/L, respectively). The low concentrations of fibrinogen in FFP make it unsuitable for fibrinogen supplementation [30,31], and the efficacy of either cryoprecipitate or Fg-C in major trauma haemorrhage remains unanswered by a randomised control trial (RCT). The results of CRYOSTAT-2, an RCT addressing whether early cryoprecipitate transfusion improves survival from major trauma haemorrhage, are eagerly awaited [22]. Cryoprecipitate contains a number of coagulation factors that are not present in Fg-C [23]; however, the large doses required to manage trauma haemorrhage can put a strain on the blood transfusion service. Fg-C has advantages including standard dose per vial, ease of transport and reduced transfusion volume.

Traditionally, fibrinogen has been supplemented only when blood levels fall below 1.5 g/L [32], but our data suggest that a much higher fibrinogen level, between 3.3 and 3.9 g/L, is required to normalise ROTEM parameters. The ROTEM CA5 parameter is used in increasing numbers of trauma centres to diagnose traumatic coagulopathy and guide transfusion therapy [33]. Our results show that high levels of fibrinogen supplementation (up to 14 g/L) continued to increase the CA5 measurement for FIBTEM and EXTEM tests, suggesting that fibrinogen continues to drive clot strength and stability at supraphysiological levels. There was no difference in ROTEM parameters between Fg-C and cryoprecipitate, and our data were therefore not able to replicate previous studies [34,35] that have found an additive effect of FXIII on clot stability above that of exogenous fibrinogen. The high levels of FXIII in cryoprecipitate have previously been shown to increase the MCF parameter when given alone [35], or in combination with fibrinogen [34]. This may be because the levels of FXIII used in this study were not supraphysiological [35].

Exogenous fibrinogen, when added to FDP, shortened standard clotting times (PT, APTT and TT) as predicted. However, significant differences were observed between cryoprecipitate and Fg-C. Increasing concentrations of cryoprecipitate continued to shorten clotting times in FDP, whereas the addition of Fg-C resulted in progressive lengthening of clotting times above 4.5 g/L. It was possible that these unexpected results were due to the presence of heparin; however, heparin is not a known constituent of Fg-C, and a Reptilase time test did not show any evidence of heparin contamination (results not shown). A possible explanation is the presence of arginine in Fg-C, a negatively charged amino acid used as an additive to aid dissolution of the fibrinogen into a solvent [36]. It is known that high concentrations of arginine inhibit thrombin activation [37], resulting in prolonged clotting times and a low coagulation factor level. Both of these effects were observed in our study at higher concentrations of Fg-C.

TG tests provide a more intricate view of coagulation capacity than conventional clotting tests [38,39,40]. We observed a significant difference between cryoprecipitate and Fg-C. The additional coagulation factors present in cryoprecipitate are likely to explain its higher thrombin generating ability. It has previously been reported that reductions in FV or FVIII lead to prolongation of the lag time and time to peak [41], and these parameters are significantly different between cryoprecipitate and Fg-C (*p* < 0.0001 and *p* < 0.05, respectively). Despite a significant difference in peak height between the two fibrinogen sources (*p* < 0.05), the ETP was not significantly different and was within the normal range for both sources. At 4.5 g/L, the ETP was 1735 and 1943 nM/min for Fg-C and cryoprecipitate, respectively. This is most likely due to the excess of fibrinogen present as a substrate. Cryoprecipitate also contains anti-coagulant factors, namely antithrombin [42], which may influence TG and explain why the ETP is comparable between the two sources.

Analysis of fibrin clot structure by confocal microscopy highlighted structural differences between clots formed with Fg-C and cryoprecipitate. Clots formed from cryoprecipitate had a more homogeneous fibrin network and higher density fibrin fibres when compared to Fg-C. Fibrin composition affects the rate of fibrinolysis; the size, number and arrangement of fibrin fibres determine the extent of tPA binding to fibrin and therefore can influence the rate of fibrinolysis [43,44,45,46,47]. Fragile clots formed from reduced numbers of fibrin fibres are implicated in bleeding disorders, such as haemophilia, where thrombi are porous and degrade faster [48,49,50,51]. Contrastingly, clots with higher fibre density make the individual more susceptible to thrombosis, and therefore cardiovascular disease, due to reduced rates of fibrinolysis [48,52,53,54].

The differences in fibrin clot structure observed in the confocal microscopy experiments were supported by the clot lysis data. Interestingly, clots formed with increasing concentrations of cryoprecipitate, but not Fg-C, had increased stability against fibrinolytic degradation (Figure 6). This suggests that the additional coagulation factors present in cryoprecipitate allow the formation of a stronger and more stable fibrin network that is resistant to premature fibrinolytic degradation. Coagulation FXIII plays a critical role in forming fibrin–fibrin and fibrin–α_2_AP cross-links [15,16,17]; therefore, it is hypothesised that the significantly higher concentration of FXIII in cryoprecipitate than Fg-C (*p* < 0.05) may alter the fibrin network. Furthermore, our data indicate that α_2_AP, which is known to prevent premature clot degradation [18], is elevated 98-fold in cryoprecipitate when compared to Fg-C (*p* < 0.001).

There are limitations to our study; firstly, the experiments were performed on FDP, which presents limitations based on its method of manufacture. FDP was used to model low fibrinogen levels observed during major haemorrhage in patients with TIC. The FDP was known to have 36%, 43% and <5% lower levels of FV, FVIII and FXIII than would be present in normal plasma. The additional factors found in cryoprecipitate may provide an advantage over Fg-C by supplementing the lower levels of FV, FVIII and FXIII in the FDP. Lower levels of FV and FVIII would certainly contribute to lower TG potential as observed in our experiments [55]. However, our results indicate that only FVIII, FXIII and vWF were significantly different at increasing concentrations of fibrinogen between cryoprecipitate and Fg-C. Lower levels of FVIII have been shown to reduce the ETP [55], and high FVIII levels are known to shorten APTT tests, but FXIII and vWF elicit little effect on standard clotting tests [56]. Whole blood or plasma obtained from an afibrinogenaemia patient would have been ideal for this study if available.

In summary, these experiments showed that standard and global clotting tests were sensitive to changes in fibrinogen concentration in our *in vitro* model of fibrinogen supplementation. Trauma coagulopathy is characterised by low fibrinogen and increased fibrinolysis. The two main sources of fibrinogen supplementation tested had comparable effects on fibrinogen recovery, but significant differences were observed with TG (likely due to FV and FVIII replacement with cryoprecipitate). Cryoprecipitate supplementation was associated with reduced susceptibility to fibrinolysis and a more homogeneous fibrin network than Fg-C. Put together, these data suggest a differential effect between cryoprecipitate and Fg-C, which may lead to differences in clot stability during trauma haemorrhage. These results need further exploration and will need evaluation in a clinical setting. We aim to answer this question using samples from trauma patients recruited to the Fibrinogen Early in Severe Trauma Study (FEISTY; NCT02745041) and randomised to receive either Fg-C or cryoprecipitate [57].

## 4. Methods

### 4.1. Fibrinogen Sources

Pooled cryoprecipitate was sourced from NHS Blood and Transplant, UK. A commercially available Fg-C, RiaSTAP, was obtained from CSL Behring, Marburg, Germany, and was reconstituted in distilled water (dH_2_O) according to the manufacturer’s instructions. Both cryoprecipitate and Fg-C were aliquoted and stored at −80 °C. PNP and FDP was obtained from Affinity Biologicals, Ontario, Canada. In some experiments, FDP was used to spike in incremental concentrations of cryoprecipitate or Fg-C. For spiking experiments, the concentration of fibrinogen in Fg-C and cryoprecipitate was determined by Clauss fibrinogen and ELISA and then diluted to the required concentration (0–15 g/L) in dH_2_O. The same batch of cryoprecipitate and Fg-C was used for all experiments.

### 4.2. Standard Laboratory Tests

The Clauss fibrinogen tests (Dade Thrombin Reagent, Siemens, Marburg, Germany), PT (Dade Innovin, Siemens, Marburg, Germany), APTT (Dade Actin FS, Siemens, Marburg, Germany) and TT (Thromboclotin, Siemens, Marburg, Germany) were processed using a Sysmex CS-5100 haematology analyser. The normal range for each test is as follows: fibrinogen (1.5–4.5 g/L), PT (13–16 s), APTT (26–36 s) and TT (16–19 s). Coagulation factors II, V, VII, VIII, IX, X, XI and XII were quantified using a Siemens Sysmex CS-5100 haematology analyser. FXIII and vWF were measured using a Siemens Sysmex CS-5100 haematology analyser and Berichrom Factor XIII Chromogenic (Siemens, Marburg, Germany) and vWF:Ag assay kit (Siemens, Marburg, Germany), respectively. Normal ranges for all coagulation factors are 50–150%. α_2_AP concentration was measured using an in-house ELISA, respectively.

### 4.3. ROTEM

ROTEM is a whole blood clotting test that assesses the viscoelastic properties of clot formation under low shear stress [58]. FDP was spiked with incremental concentrations of fibrinogen from either Fg-C or cryoprecipitate sources. EXTEM (tissue factor (TF) activator to evaluate extrinsic pathway) and FIBTEM (to evaluate fibrinogen contribution by inhibition of platelets with cytochalasin D) tests were performed on all samples. The CT, alpha angle, CA5 and MCF were exported from the TEMogram for statistical analysis.

### 4.4. Thrombin Generation

TG was performed on FDP spiked with incremental concentrations of fibrinogen from either Fg-C or cryoprecipitate sources. TG was triggered with 5 pM TF and 4 µM phospholipids in the presence of a fluorogenic substrate and CaCl_2_ (Diagnostica Stago, Asnieres, France). TG was measured using the calibrated automated thrombogram (CAT) [59] and thrombinoscope v5 software. The lag time, time to peak, peak height and ETP parameters were extracted from the thrombogram and exported for statistical analysis.

### 4.5. Confocal Microscopy

Clots were formed from 30 % FDP, 16 µM phospholipids (Rossix, Molndal, Sweden) and 0.25 µM AlexaFluor 488 labelled fibrinogen (ThermoFisher Scientific, Waltham, USA) in 10 mM Tris pH 7.4, 140 mM NaCl, 0.01% TWEEN-20. FDP was substituted with PNP for a control. Cryoprecipitate and Fg-C were added at a range of concentrations—0.5, 2 and 3 mg/mL. Clotting was initiated by the addition of 0.1 U/mL thrombin (Sigma Aldrich, St Louis, MS, USA) and 10.6 mM CaCl_2_. Clots were polymerised in an Ibidi μ-slide VI^0.4^ chamber and incubated for 2 h at 37 °C in a moist box. Clots were imaged using a ×63 1.4 oil immersion objective and Zeiss 710 laser scanning confocal microscope. Images were recorded on differential interference contrast (DIC) microscopy and at excitation wavelengths of 488 nm and analysed using Zen 2012 SP1 v8.1 (Black edition).

### 4.6. Clot Lysis

FDP (30% total volume), 16 µM phospholipids (Rossix, Molndal, Sweden) and 300 pM tPA (NIBSC, Potters Bar, UK) in 10 mM TRIS pH 7.4 0.01% Tween20 were added to 96 well flat-bottom assay plates. A range of fibrinogen concentrations, 0.5–3 mg/mL, were added from either Fg-C or cryoprecipitate. FDP was substituted with PNP for a control. Clotting was initiated with 0.01 U/mL thrombin (Sigma Aldrich, St Louis, USA) 10.6 mM CaCl_2_ and clot formation and lysis monitored using a Labsystems iEMS plate reader. Absorbance at 405 nm was recorded every 60 s for 4 h using Ascent software (version 2.6). Data were analysed by calculating time to 50% lysis using Shiny App software for clot lysis (2019 version) [25].

### 4.7. Data Analysis

Results are represented by the mean ± standard deviation (SD). The number of repeats for the clot lysis and confocal microscopy was ≥3 and experiments were performed in duplicate on different days. For the remaining assays, the number of repeats was 2, and experiments were performed in duplicate (coagulation testing/factors) or triplicate (ROTEM and TG) on two different days. In this case, the number of repeats was lower to ensure all repeats used the same batch of cryoprecipitate and Fg-C. Statistical analysis was performed using Graph Pad Prism v8.4 (California, CA, USA) and normality assessed using a D’Agostino–Pearson omnibus test. A non-parametric Mann–Whitney *t*-test was used to analyse the data. *p* < 0.05 was considered significant.

## Figures and Tables

**Table 1:** Constituents of fibrinogen sources.

	PNP	FDP	Cryoprecipitate	Fg-C
**Clauss Fg (g/L)**	3.3 ± 0.3	<0.15	6.8 ± 0.1	21.6 ± 0.1
**FII (%)**	98 ± 4	98 ± 7	101 ± 12	<1
**FV (%)**	82	36 ± 0.7	68 ± 9	<1
**FVII (%)**	84 ± 1	75 ± 4	81 ± 7	<1
**FVIII (%)**	107 ± 14	43 ± 5	190 ± 0.6	<1
**FIX (%)**	127 ± 25	106 ± 3	105 ± 8	2
**FX (%)**	92 ± 7	95 ± 3	98 ± 14	<1
**FXI (%)**	100 ± 16	107 ± 4	92 ± 3	<1
**FXIII (%)**	80 ± 7	<5	105 ± 3	<1
**vWF:Ag (%)**	127 ± 33	65 ± 1	288 ± 66	66
**α_2_AP (µg/mL)**	72 ± 20	38 ± 3	98 ± 8	1 ± 3

Clauss fibrinogen and coagulation factors II, V, VII, VIII, IX, X, XI, XIII and vWF antigen were quantified using a Sysmex CS-5100 haematology analyser in pooled normal plasma (PNP), fibrinogen-deficient plasma (FDP), cryoprecipitate and fibrinogen concentrate (Fg-C). Results are represented by the mean ± SD and expressed as a percentage (%) of normal, except for Clauss fibrinogen which is reported as a concentration (g/L). The normal range for all factor assays is 50–150% and Clauss fibrinogen 1.5–4.5 g/L. *n* = 2. α_2_AP levels were quantified using an in-house enzyme-linked immunosorbent assay (ELISA). *n* = 7.

## Data Availability

Data sharing not applicable.
